# Usefulness of panoramic 344°-viewing in Crohn’s disease capsule endoscopy: a proof of concept pilot study with the novel PillCam™ Crohn’s system

**DOI:** 10.1186/s12876-020-01231-0

**Published:** 2020-04-07

**Authors:** Gian Eugenio Tontini, Fernando Rizzello, Flaminia Cavallaro, Gianluca Bonitta, Dania Gelli, Luca Pastorelli, Marco Salice, Maurizio Vecchi, Paolo Gionchetti, Carlo Calabrese

**Affiliations:** 1 Gastroenterology and Endoscopy Unit, Fondazione IRCCS Ca’ Granda Ospedale Maggiore Policlinico, Milan, Italy; 2 Department of Pathophysiology and Transplantation, University of Milan, Milan, Italy; 3 IBD Unit, Department of Medical and Surgical Sciences (DIMEC), Policlinico S.Orsola-Malpighi, University of Bologna, Bologna, Italy; 4 Gastroenterology and Digestive Endoscopy Unit, IRCCS Policlinico San Donato, San Donato Milanese, Milan, Italy; 5 Department of Biomedical Sciences for Health, University of Milan, Milan, Italy

**Keywords:** Capsule endoscopy, Crohn’s disease, Enteroscopy, Inflammatory bowel disease, Pan-intestinal endoscopy, PillCam™ Crohn’s system

## Abstract

**Background:**

A new capsule endoscopy (CE) system featuring two advanced optics for 344°-viewing and a prolonged operative time has been recently developed for Crohn’s disease (CD) patients. Hence, we evaluated, for the first time, the performance of this novel CE and the add-on value of the 344°-viewing in a multi-center real-life setting.

**Methods:**

Consecutive patients with suspected or established CD received the PillCam™ Crohn’s System as supplementary diagnostic work-up focused on the small-bowel between June 2017 and June 2018. Technical and clinical data, including the panenteric CE diagnostic yield, the Lewis score and the impact of small-bowel findings on clinical management during a 6-months follow-up (new diagnosis, staging or treatment upgrade) were collected, thereby evaluating the added value of the 344° panoramic-view (lesions detected by camera A and B) over the standard 172°-view (lesions detected by one camera only).

**Results:**

Among 41 patients (aged 43 ± 20 years), 73% underwent CE for suspected CD and 27% for established CD. The rate of complete enteroscopy was 90%. No technical failure or retention occurred. Compared to the standard 172° view, the panoramic 344°-view revealed a greater number of patients with a relevant lesion (56.1% vs. 39.0%; *P* = 0.023), resulting in higher Lewis score (222,8 vs. 185.7; *P* = 0.031), and improved clinical management (48.8% vs. 31.7%, *P* = 0.023).

**Conclusions:**

The panoramic 344°-view increases small-bowel CE accuracy, thereby improving the clinical management of CD patients with mild small-bowel active disease. This system should be regarded as a new standard for both small-bowel diagnosis and monitoring in inflammatory bowel diseases.

## Background

Capsule endoscopy (CE) is the most sensitive diagnostic tool for the diagnosis of small-bowel mucosal lesions in Crohn’s disease (CD) patients [1–4]. Furthermore, CE has emerged as a key tool for disease staging (i.e. location, extent, severity) and monitoring, facilitating a refined clinical classification and tight control management [5–8].

In recent years, a colonic capsule with an extended battery life and cameras on both sides has been adapted for use in inflammatory bowel diseases (IBD) as a mini-invasive approach for panenteric endoscopy in post-operative follow-up, children and special situations [9–11].

In 2017 a new capsule system designed for CD patients was released (PillCam™ Crohn’s System, PCS; Medtronic, Dublin, Ireland) [11, 12]. The PCS capsule is equipped with two advanced optics enabling a 344°-wide view between both capsule heads and with a prolonged operative time (≥12 h) to provide the panoramic visualization of the entire GI tract within a single endoscopic procedure. PCS includes a renewed software (Rapid 9) for capsule localization and dedicated applications to classify disease severity and extent according to structured descriptors or the Lewis score (LS) [11, 12]. The aim of the present multi-center study was: firstly to evaluate for the first time the performances of the novel PillCam™ Crohn’s system (PCS) in a multi-center clinical practice setting; secondly, to determine whether the 344°-panoramic view provided by its novel dual-ended camera can improve the small-bowel detection rates and the assessment of the Lewis score, thereby resulting in a different clinical management of CD patients.

## Methods

### Study population and CE procedure

All consecutive patients undergoing CE for suspected or established CD were enrolled at two Italian IBD centers between June 2017 and June 2018. According to the current guidelines and international consensus, patients with “suspected CD” were defined by the presence of suspicious symptoms of active small-bowel CD (i.e. abdominal pain, diarrhoea and weight loss) following inconsistent findings at ileocolonoscopy and esophagogastroduodenoscopy plus either extra-intestinal manifestations, inflammatory markers (i.e. C-reactive protein and faecal calprotectin), or abnormal imaging studies [1, 2, 13]. Patients with known CD underwent CE for either disease staging or monitoring following upper and/or lower endoscopy as a part of routine diagnostic work-up [1–3]. Cross-sectional imaging or patency capsule were performed in established CD and in selected patients with suspected CD [1–3].

All CE procedures were conducted using the novel novel PCS. This system includes a dual-camera capsule device with prolonged battery life potentially allowing for pan-enteric wireless capsule endoscopy and an optimized platform specifically designed to assess IBD mucosal disease severity and extent over time. Technical details of the PCS have been recently described in detail elsewhere [14].

All the patients provided their informed consent, followed a clear liquid diet for 24 h plus 12 h fasting and received 2 L of polyethylene glycol solution 2–8 h before capsule ingestion according to the local center protocols for small-bowel cleansing.

### Technical and clinical outcomes

For each procedure, technical (e.g. transit and operative times, technical failures, small-bowel completion rate), clinical (e.g. endoscopic findings, Lewis Score, capsule impact on clinical management) and safety (e.g. capsule retention and aspiration) data were collected by structured data entry. All the endoscopic findings detected along the digestive tract were systematically classified according to location (i.e. upper GI, three small-bowel tertiles, colon) and endoscopic relevance, after reaching a perfect agreement between two expert capsule readers.

Consistent with the literature, the presence of diffuse edema, ulcers, and strictures were defined as “relevant endoscopic findings for the diagnosis of active CD” [15].

The Lewis Score (LS) was calculated to assess the small-bowel CD diagnosis and activity. Briefly, the LS uses the CE structured terminology (edema, ulcer, and strictures) to grade disease activity into three levels: i) no or clinically insignificant (LS < 135); ii) mild (135 ≤ LS ≤790); iii) moderate to severe (LS > 790) [14, 15]. Peptic disorders, vascular and inflammatory lesions were assessed following established classifications [16–18].

During a 6-months follow-up, the “impact of CE on clinical management” was defined as positive when strongly supporting a definite diagnosis for patients with suspected CD, as well as when leading to refined disease staging or treatment strategy upgrade for established CD patients. Since all patient had previously received colonoscopy, only small bowel findings were considered when assessing the impact of CE on clinical management. A “definite diagnosis of CD” was defined in patients with relevant capsule endoscopy findings for the diagnosis of active CD plus coherent device-assisted enteroscopy with histopathology or cross-sectional imaging during follow-up [3]. A “treatment strategy upgrade” was considered for patients receiving treatment (add on or switch to) steroids, immunomodulators or biologics during follow-up [5, 6, 8].

Four expert capsule readers with a substantial clinical and endoscopic background in the IBD field were involved (GET, FC, MS, CC). Firstly, the capsule records were evaluated by two independents readers to define the exact location and endoscopic relevance of each endoscopic finding. Then, the capsule readers reviewed separately the two capsule camera recordings to ascertain whether each relevant endoscopic finding was clearly identified by either capsule camera A or B or both. In order to prevent findings duplication, the endoscopic findings presenting with an identical location and similar features (morphology, size) were accounted as a unique lesion. A perfect agreement between the 2 independents reviewers was required before data entry.

### Data analysis

The rate of patients with at least one relevant endoscopic finding throughout the GI tract (i.e., panenteric diagnostic yield), the Lewis Score and the impact of small-bowel findings on clinical management were separately calculated for the capsule camera recording A + B and capsule camera recording A to measure the added value of the 344°-wide panoramic-view (capsule camera A and B) over the standard CE 172°-wide view (one capsule camera only).

The descriptive statistics were presented as mean ± SD for continuous variables and percentages for categorical variables. Categorical variables were analyzed by the McNemar test, while continuous variables by the Wilcoxon test.

This study was carried out in accordance with the Declaration of Helsinki adopted in 1964 incorporating all later amendments.

## Results

Overall, 41 patients (16 men; age: 43 ± 20 years) were consecutively enrolled; 73% (30/41) of them underwent CE for suspected CD, while 27% (11/41) for established CD with a mean time of 12 years elapsed from diagnosis (Table 1).

**Table 1:** Study population - demographic and clinical data

	Study population (***n*** = 41)	Suspected CD (***n*** = 30)	Established CD (***n*** = 11)
Gender (Women / Men)	25 / 16	21 / 9	4 / 7
Age (median ± SD, years)	43 ± 20	39 ± 22	37 ± 11
Years from diagnosis (median ± SD, years)	/	/	10 ± 11
Baseline Montreal classification L1 / L2 / L3	/	/	7 / 3 / 1
Baseline Montreal classification B1 / B2 + B3	/	/	11 / 0
Completion rate^a^	90.2%	93.3%	81.8%
Positive capsule / Lewis score (median ± SD)^ba^	23 / 222,8 ± 347.1	14 / 107 ± 162	9 / 580 ± 516

^a^Incomplete enteroscopy occurred due to ulcerated narrowings (2 in suspected and 1 in established CD) and diffuse inflammation (1 case in established CD)

^b^One CE was positive for duodenal peptic disease in the suspected CD group; this case was not included in the Lewis score calculation

All the patients with established CD had a negative patency test, while no suspected CD ones received a patency capsule. No technical failure, nor capsule retention or aspiration occurred.

The mean gastric transit time, small-bowel transit time and operative time were respectively 34 ± 31 min, 264 ± 221 min and 11.8 ± 3.3 h.

Small-bowel visibility was graded as good to optimal, while colon visibility as poor to fair in all the procedures.

The rate of complete enteroscopy was 90%; incomplete enteroscopy occurred due to ulcerated narrowings (2 cases in suspected, 1 in established CD) and diffuse inflammation (1 case in established CD). As far as the Lewis score calculation is concerned, incomplete enteroscopies were excluded from the Lewis score analysis.

**Fig. 1:**
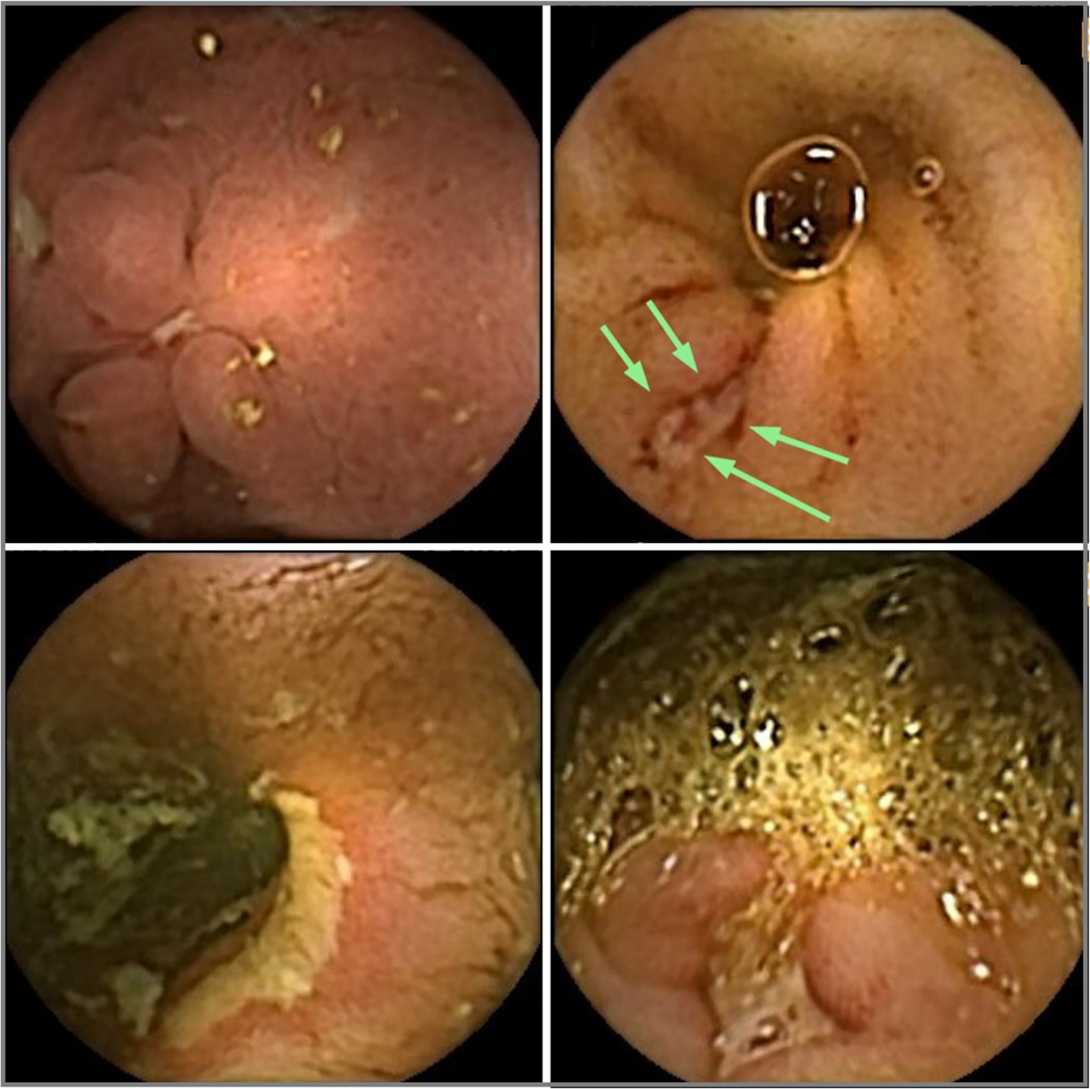
Top left, proximal duodenitis with aphthous ulcers. Top right, an isolated ulcer, 8 mm in size, identified only by one in the terminal ileum. Lower right, a deep ulcer, 10 mm in size, in the right colon. Lower left, jejunal narrowing with a large, superficial ulcer

Overall, 122 lesions were found; of them, 55 (45.1%) were classified as relevant and distributed in duodenum (10.9%), jejunum (32.7%), ileum (43.6%) or colon (12.7%) (Fig. 1). The patients with one or more panenteric relevant lesions were 23/41 (56.1%): 46.7% of those who performed CE for suspected CD and 81.8% for established CD. Notably, when diffuse edema was accounted as a relevant lesion, at least one additional and harder capsule finding has been also clearly identified, including ulcer and/or stricture.

Within the suspected-CD group with relevant lesions at CE, all detected lesions were confined to the small-bowel and one patient had an isolated duodenal ulcer, which had been missed by previous upper endoscopy and even diagnosed as a peptic disorder.

Overall, the mean Lewis score was 222,8 ± 347.1. Patients with suspected CD had a mean Lewis score of 107 ± 162, while those with established CD had a mean Lewis score of 580 ± 516. Overall, 51.4% of patients had Lewis score ≥ 135 (39.3% in suspected CD, 77.7% in established CD patients).

During a 6-month follow up, the small bowel finding resulting from CE investigation had an impact on the clinical management in 48.8% of patients, leading to the final diagnostic definition for 43.3% of patients with suspected CD (12 new diagnosis of CD and one peptic disease) and to disease staging upgrade (2 patients), treatment escalation (4 patients) or both (one patient) for 63.6% of those with established CD (Table 2).

**Table 2:** Impact of standard and panoramic viewing in suspected and established CD patients

	One Camera (172°-view)	Two cameras (344°-view)
Lewis Score (overall population)	185.7	222.8
Lewis Score in suspected CD	97	107
Lewis Score established CD	442	580
New diagnosis of CD^a^	10/30	13/30
Montreal class upgrade	1/11	3/11
Treatment strategy upgrade	3/11	5/11

^a^ New diagnosis of CD was defined for relevant CE findings strongly supporting a definite diagnosis following a 6-months follow-up

As compared to the standard 172° view, the panoramic 344°-view detected 17.1% more patients with at least one relevant lesion (56.1% vs. 39.0%; *P* = 0.023; Video 1), resulting in a 16.6% increase of the mean LS (222.8 vs. 185.7; *P* = 0.031), and a 17.1% increase in the number of patients with improved clinical management (48.8% vs. 31.7%, *P* = 0.023) (Table 2).


**Additional file 1: Video 1.** PillCam Crohn’s record of the terminal ileum showing an isolated ulcer, partially depressed, 8 × 4 mm in size, identified only by one camera.


## Discussion

This observational multi-center study represents the first series of consecutive patients undergoing CE with the novel PCS in a clinical practice setting. This system features two independent optics placed on both capsule edges providing a 344°-wide panoramic-view to improve endoscopic detection and characterization throughout the entire GI tract [11, 19]. A recent pilot study has suggested that PCS would possibly result in higher diagnostic yields than ileo-colonoscopy in patients with known active CD following a capsule colon cleansing protocol [12]. PCS showed to be safe and to enable the direct evaluation of the entire gut based on the preliminary data from a proof-of-concept feasibility study conducted on CD and ulcerative colitis patients [14].

Our study confirms the high technical and diagnostic performances of the PCS in patients with suspected or established CD. No safety nor technical issues were observed, the small-bowel completion rate was > 90% and the capsule operating time was 11.8 ± 3.3 h, thereby largely fitting with the panenteric assessment [9–11]. In fact, PCS identified several lesions in the duodenum and colon, confirming the potential of the 344°-wide panoramic view as a first triage tool for panenteric endoscopy in IBD.

However, the present observational study was carried out in a real life setting where CE examinations are performed as a supplementary diagnostic work-up on the small-bowel mucosa, thereby avoiding the large bowel cleansing. As a result, PCS revealed relevant small-bowel findings supporting the new diagnosis of CD (LS ≥ 135) in one-third of the patients with suspected CD. In established CD patients, the recognition of active small-bowel disease resulted in refined disease staging and targeted treatment escalation, thereby strengthening the emerging role of CE in two-thirds of cases [5–7]. These results are consistent with those reported in two longitudinal studies conducted with standard-view CE devices showing an impact on clinical management in 34–52% of established CD patients [6, 8].

No previous study has ever assessed whether the peculiar 344°-wide panoramic-view of the novel PCS can offer any advantage in clinical practice over the standard 172°-view even in the small-bowel. Consistently, we have performed a post hoc revision of all PCS video records to ascertain whether each relevant endoscopic finding was clearly identified by either capsule camera A or B. Then, we compared the diagnostic outcomes and the contribution to the clinical outcomes resulting from standard 172°-view (one camera only) and 344°-wide panoramic view (camera A and B). Surprisingly, the panenteric CE diagnostic yield and the Lewis score resulted remarkably enhanced with the 344°-wide panoramic view. More interestingly, 344°-wide panoramic view resulted in a Δ + 17% examinations with a real impact on patients’ clinical management. This initial evidence suggests the PCS as a more accurate and effective device for small bowel CE in IBD patients. Further studies should now confirm this figure in larger cohorts of patients stratified according to the severity of the small-bowel involvement. In fact, the predominance of mild to moderate active disease observed in our population (mean LS = 222,8 ± 347.1) could have highlighted the add-on value of the panoramic 344°-viewing CE, which is supposed to be less relevant in severe CD enteropathies.

Potential limitations of the present study must be addressed. Firstly, given the observational design, our capsule readers were not blinded either to previous investigations or to the yield of the “single-view” camera. Secondly, despite the exact number and location of findings appears substantial using the renewed Rapid 9 software for capsule localization, anterograde and retrograde movements may result in an unbalanced overestimation of detected lesions between the 172° and 344° capsule view. Thirdly, according to the current European guidelines on IBD [1–3], CE was performed as a supplementary diagnostic work-up following a previous ileo-colonoscopy. Therefore, no colon cleansing protocol was performed before CE to avoid the double administration of substantial purgative agents, thereby reducing the diagnostic potential of PCS into the colon. In addition, two little shortcomings related to the use of the PCS should be also mentioned. The dual camera-viewing implies additional images to be downloaded and assessed. This might increase the reading time of about 5–10 min even if the operator assesses the two cameras simultaneously in dual mode. The present version of the PCS provides a lower image quality as compared to the PillCam SB3 [20].

## Conclusions

This proof of concept pilot multi-center study carried out in routine clinical practice confirms that the novel PillCam™ Crohn’s system is safe and provides high technical and endoscopic performances for CD assessment [12, 14]. Our results also suggest that the panoramic 344°-view clearly improves small-bowel capsule visualisation, thus potentially improving both diagnostic accuracy and clinical management for patients undergoing small bowel CE for suspected or established CD.

## Data Availability

The datasets generated during the current study are available from the corresponding author on reasonable request.

## Abbreviations

CE: Capsule endoscopy
CD: Crohn’s disease
IBD: Inflammatory bowel diseases
LS: Lewis score
PCS: PillCam™ Crohn’s System

## References

[1] Annese V, Daperno M, Rutter MD, Amiot A, Bossuyt P, East J, Ferrante M, Götz M, Katsanos KH, Kießlich R, Ordás I, Repici A, Rosa B, Sebastian S, Kucharzik T, Eliakim R (2013). European evidence-based consensus for endoscopy in inflammatory bowel disease. J Crohns Colitis.

[2] Pennazio M, Spada C, Eliakim R, Keuchel M, May A, Mulder CJ, Rondonotti E, Adler SN, Albert J, Baltes P, Barbaro F, Cellier C, Charton JP, Delvaux M, Despott EJ, Domagk D, Klein A, McAlindon M, Rosa B, Rowse G, Sanders DS, Saurin JC, Sidhu R, Dumonceau JM, Hassan C, Gralnek IM (2015). Small-bowel capsule endoscopy and device-assisted enteroscopy for diagnosis and treatment of small-bowel disorders: European Society of Gastrointestinal Endoscopy (ESGE) clinical guideline. Endoscopy.

[3] Gomollón F, Dignass A, Annese V, Tilg H, Van Assche G, Lindsay JO, Peyrin-Biroulet L, Cullen GJ, Daperno M, Kucharzik T, Rieder F, Almer S, Armuzzi A, Harbord M, Langhorst J, Sans M, Chowers Y, Fiorino G, Juillerat P, Mantzaris GJ, Rizzello F, Vavricka S, Gionchetti P, ECCO (2017). 3rd European evidence-based consensus on the diagnosis and management of Crohn’s disease 2016: part 1: diagnosis and medical management. J Crohns Colitis.

[4] Kopylov U, Yung DE, Engel T, Vijayan S, Har-Noy O, Katz L, Oliva S, Avni T, Battat R, Eliakim R, Ben-Horin S, Koulaouzidis A (2017). Diagnostic yield of capsule endoscopy versus magnetic resonance enterography and small bowel contrast ultrasound in the evaluation of small bowel Crohn’s disease: systematic review and meta-analysis. Dig Liver Dis.

[5] Cotter J, Dias de Castro F, Moreira MJ, Rosa B (2014). Tailoring Crohn’s disease treatment: the impact of small bowel capsule endoscopy. J Crohns Colitis.

[6] Kopylov U, Nemeth A, Koulaouzidis A, Makins R, Wild G, Afif W, Bitton A, Johansson GW, Bessissow T, Eliakim R, Toth E, Seidman EG (2015). Small bowel capsule endoscopy in the management of established Crohn’s disease: clinical impact, safety, and correlation with inflammatory biomarkers. Inflamm Bowel Dis.

[7] González-Suárez B, Rodriguez S, Ricart E, Ordás I, Rimola J, Díaz-González Á, Romero C, de Miguel CR, Jáuregui A, Araujo IK, Ramirez A, Gallego M, Fernández-Esparrach G, Ginés Á, Sendino O, Llach J, Panés J (2018). Comparison of capsule endoscopy and magnetic resonance enterography for the assessment of small bowel lesions in Crohn’s disease. Inflamm Bowel Dis.

[8] Hansel SL, McCurdy JD, Barlow JM, Fidler J, Fletcher JG, Becker B, Prabhu NC, Faubion WA, Hanson KA, Kane SV, Kisiel JB, Loftus EV, Papadakis KA, Pardi DS, Raffals LE, Schoenoff S, Tremaine WJ, Bruining DH (2018). Clinical benefit of capsule. Endoscopy in Crohn’s disease: impact on patient management and prevalence of proximal small bowel involvement. Inflamm Bowel Dis.

[9] Oliva S, Cucchiara S, Civitelli F, Casciani E, Di Nardo G, Hassan C, Papoff P, Cohen SA (2016). Colon capsule endoscopy compared with other modalities in the evaluation of pediatric Crohn’s disease of the small bowel and colon. Gastrointest Endosc.

[10] Hausmann J, Schmelz R, Walldorf J, Filmann N, Zeuzem S, Albert JG (2017). Pan-intestinal capsule endoscopy in patients with postoperative Crohn’s disease: a pilot study. Scand J Gastroenterol.

[11] Carvalho PB, Rosa B, Dias de Castro F, Moreira MJ, Cotter J (2015). PillCam COLON 2© in Crohn’s disease: a new concept of pan-enteric mucosal healing assessment. World J Gastroenterol.

[12] Leighton JA, Helper DJ, Gralnek IM, Dotan I, Fernandez-Urien I, Lahat A, Malik P, Mullin GE, Rosa B (2017). Comparing diagnostic yield of a novel pan-enteric video capsule endoscope with ileocolonoscopy in patients with active Crohn’s disease: a feasibility study. Gastrointest Endosc.

[13] Mergener K, Ponchon T, Gralnek I, Pennazio M, Gay G, Selby W, Seidman EG, Cellier C, Murray J, de Franchis R, Rösch T, Lewis BS (2007). Literature review and recommendations for clinical application of small-bowel capsule endoscopy, based on a panel discussion by international experts. Consensus statements for small-bowel capsule endoscopy, 2006/2007. Endoscopy.

[14] Eliakim R, Spada C, Lapidus A, Eyal I, Pecere S, Fernández-Urién I, Lahat A, Costamagna G, Schwartz A, Ron Y, Yanai H, Adler S (2018). Evaluation of a new pan-enteric video capsule endoscopy system in patients with suspected or established inflammatory bowel disease - feasibility study. Endosc Int Open.

[15] Gralnek IM, Defranchis R, Seidman E, Leighton JA, Legnani P, Lewis BS (2008). Development of a capsule endoscopy scoring index for small bowel mucosal inflammatory change. Aliment Pharmacol Ther.

[16] Forrest JA, Finlayson ND, Shearman DJ (1974). Endoscopy in gastrointestinal bleeding. Lancet.

[17] Leenhardt R, Li C, Koulaouzidis A, Cavallaro F, Cholet F, Eliakim R, Fernandez-Urien I, Kopylov U, McAlindon M, Németh A, Plevris JN, Rahmi G, Rondonotti E, Saurin J-C, Tontini GE, Toth E, Yung D, Marteau P, Dray X (2019). Nomenclature and semantic description of vascular lesions in small bowel capsule endoscopy: an international Delphi consensus statement. Endosc Int Open.

[18] Leenhardt R, Buisson A, Bourreille A, Marteau P, Koulaouzidis A, Li C, Keuchel M, Rondonotti E, Toth E, Plevris JN, Eliakim R, Rahmi G, Saurin JC, Dray X, Rosa B, Triantafyllou K, Elli L, Wurm Johansson G, Panter S, Ellul P, Robles EP-C, McNamara D, Beaumont H, Spada C, Cavallaro F, Cholet F, Fernandez-Urien Sainz I, Kopylov U, McAlindon ME, Németh A, Tontini GE, Yung D, Niv Y, the ESGE small-bowel research group (2020). Nomenclature and semantic description of ulcerative and inflammatory lesions seen in Crohn’s disease in small bowel capsule endoscopy: an international Delphi consensus statement. United European Gastroenterol J.

[19] Tontini GE, Wiedbrauck F, Cavallaro F, Koulaouzidis A, Marino R, Pastorelli L, Spina L, McAlindon ME, Leoni P, Vitagliano P, Cadoni S, Rondonotti E, Vecchi M (2017). Small-bowel capsule endoscopy with panoramic view: results of the first multicenter, observational study (with videos). Gastrointest Endosc.

[20] Tontini GE, Manfredi G, Orlando S, Neumann H, Vecchi M, Buscarini E, Elli L (2019). Endoscopic ultrasonography and small-bowel endoscopy: present and future. Dig Endosc.

